# Supplementary material for: Radiogenomic signatures reveal multiscale intratumour heterogeneity associated with biological functions and survival in breast cancer
Source: Nat Commun. 2020 Sep 25;11:4861. doi: 10.1038/s41467-020-18703-2 (PMC7519071; doi:10.1038/s41467-020-18703-2)
Supplement: Supplementary file 1 — Supplementary Information [file 41467_2020_18703_MOESM1_ESM.pdf]

Radiogenomic signatures reveal multiscale intratumour heterogeneity associated with biological functions and survival in breast cancer

Fan et al.

Supplementary Table 1. Pathway analysis for the subclone-specific marker genes

Term	P-Value	Corrected P
**Subclone 1**		
**Cell cycle**	**2.45E-12**	**1.45E-10**
DNA replication	1.11E-08	3.28E-07
Progesterone-mediated oocyte maturation	1.19E-06	2.34E-05
Oocyte meiosis	3.48E-06	5.13E-05
p53 signaling pathway	0.000296	0.003497
Mismatch repair	0.000915	0.008167
Pyrimidine metabolism	0.000969	0.008167
Alanine, aspartate and glutamate metabolism	0.002004	0.014201
Apoptosis	0.002166	0.014201
Hepatitis B	0.002434	0.014361
Nucleotide excision repair	0.00349	0.018719
Fanconi anemia pathway	0.004693	0.023074
Small cell lung cancer	0.010863	0.042706
Gap junction	0.01134	0.042706
HTLV-I infection	0.011574	0.042706
Ribosome biogenesis in eukaryotes	0.011581	0.042706
**Subclone 2**		
**Alzheimer's disease**	0.000196488	0.017880403
Nicotinate and nicotinamide metabolism	0.001299193	0.032920243
Oxidative phosphorylation	0.001522983	0.032920243
Apoptosis	0.00175785	0.032920243
Parkinson's disease	0.001828869	0.032920243
Non-alcoholic fatty liver disease (NAFLD)	0.002170565	0.032920243
Amyotrophic lateral sclerosis (ALS)	0.003529475	0.03602332
Endometrial cancer	0.00366132	0.03602332
Non-small cell lung cancer	0.004211341	0.03602332
Huntington's disease	0.004277433	0.03602332
Acute myeloid leukemia	0.004354467	0.03602332
VEGF signaling pathway	0.004949232	0.037531672
Melanoma	0.006589326	0.045130346
Chronic myeloid leukemia	0.00694313	0.045130346
**Subclone 3**		
**Peroxisome**	1.55E-05	0.001057
Prostate cancer	0.000536	0.011752
Pathways in cancer	0.000632	0.011752
Vitamin digestion and absorption	0.000909	0.011752
Metabolic pathways	0.00129	0.011752

Oocyte meiosis	0.001333	0.011752
Mucin type O-Glycan biosynthesis	0.001466	0.011752
beta-Alanine metabolism	0.001466	0.011752
Propanoate metabolism	0.001555	0.011752
Wnt signaling pathway	0.002031	0.013814
Hedgehog signaling pathway	0.003207	0.01891
Valine, leucine and isoleucine degradation	0.003337	0.01891
Basal cell carcinoma	0.004315	0.022417
Staphylococcus aureus infection	0.004615	0.022417
Pertussis	0.007738	0.03508
Complement and coagulation cascades	0.008531	0.036258
HTLV-I infection	0.010297	0.041189
**Subclone 4**		
**ECM-receptor interaction**	8.77E-05	0.003509
Focal adhesion	0.001172	0.023447
Arrhythmogenic right ventricular cardiomyopathy (ARVC)	0.002678	0.031149
Hypertrophic cardiomyopathy (HCM)	0.003335	0.031149
Dilated cardiomyopathy	0.003894	0.031149
PI3K-Akt signaling pathway	0.005032	0.033543
**Subclone 5**		
**Cholinergic synapse**	0.00026	0.010569
Neurotrophin signaling pathway	0.000325	0.010569
Oocyte meiosis	0.000348	0.010569
cGMP-PKG signaling pathway	0.000833	0.018955
Regulation of lipolysis in adipocytes	0.001813	0.024216
Longevity regulating pathway - multiple species	0.00234	0.024216
Long-term potentiation	0.002482	0.024216
Thyroid hormone synthesis	0.002854	0.024216
Gastric acid secretion	0.003088	0.024216
Aldosterone synthesis and secretion	0.00367	0.024216
Gap junction	0.004299	0.024216
Salivary secretion	0.004392	0.024216
GnRH signaling pathway	0.004582	0.024216
Longevity regulating pathway	0.004874	0.024216
Circadian entrainment	0.004973	0.024216
Pancreatic secretion	0.005074	0.024216
Endocrine resistance	0.005175	0.024216
Inflammatory mediator regulation of TRP channels	0.005277	0.024216
Progesterone-mediated oocyte maturation	0.005277	0.024216
Estrogen signaling pathway	0.00538	0.024216

Retrograde endocannabinoid signaling	0.005588	0.024216
Glutamatergic synapse	0.007033	0.029091
Vascular smooth muscle contraction	0.007751	0.030323
Platelet activation	0.007997	0.030323
Apoptosis	0.010371	0.037749
Adrenergic signaling in cardiomyocytes	0.01166	0.040811
Oxytocin signaling pathway	0.013017	0.043871
Alzheimer's disease	0.0146	0.047451
**Subclone 6**		
Primary immunodeficiency	1.19E-10	5.13E-09
Hematopoietic cell lineage	7.11E-09	1.53E-07
Measles	3.26E-06	4.67E-05
HTLV-I infection	3.89E-05	0.000365
Cytokine-cytokine receptor interaction	4.25E-05	0.000365
T cell receptor signaling pathway	6.75E-05	0.000484
Natural killer cell mediated cytotoxicity	0.00014	0.000858
Jak-STAT signaling pathway	0.00022	0.001182
B cell receptor signaling pathway	0.001377	0.00658
PI3K-Akt signaling pathway	0.001998	0.008584
Fc gamma R-mediated phagocytosis	0.002196	0.008584
Chagas disease (American trypanosomiasis)	0.002723	0.009759
Cell adhesion molecules (CAMs)	0.005224	0.017278
Endocytosis	0.0156	0.047913
**Subclone 7**		
Protein digestion and absorption	2.74E-05	0.000411
Nitrogen metabolism	6.44E-05	0.000483

Note: Corrected p values less than 0.05 are shown. Statistical significance was assessed by the two-sided hypergeometric test. *p* values were adjusted for multiple comparisons using Benjamini-Hochberg method.

Supplementary Table 2. Imaging features in multivariate predictive model for subclone compositions

Tumour	Tumour surrounding Pachynema
Entropy[1]	Maximum[1]
Minimum[3]	Maximum Probability[1]
Correlation[1]	Contrast[2]
Entrophy[2]	Surface Area/Volume
Sum Entropy[2]	Mean Radius of Lesion
Maximum Probability[1]	Compactness[1]
Surface Area/Volume	
Maximum Lesion Radius[1]	
Compactness[1]	

[1]precontrast image

[2]image subtractions between postcontrast image series and precontrast series

[3]image subtractions between late postcontrast and precontrast series

Supplementary Table 3. Demographic and clinical data of the study dataset

Parameter	Genomic development dataset (n =660)	Genomic validation dataset (n =329)	Radiogenomic dataset (n =87)	Prognostic assessment (n=173)	Prognostic validation (n=61)
**Age (y)**					
Median	59 (26-90)	58 (26-90)	53 (29-82)	47.8 (26.7-68.8)	48 (29.7-72.4)
Mean ± STD	58.7±12.9	58.7±13.9	53.3±11.5	47.7± 8.8	48.1±9.8
**Race**					
Asian	45 (7)	14 (4)	0	7 (4)	3 (5)
Black or African American	118 (18)	59 (18)	4 (5)	30 (17)	3 (5)
White	442 (67)	227 (69)	82 (94)	135 (78)	47 (77)
Unknown or others	55 (8)	29 (9)	1 (1)	1 (1)	8 (13)
**ER**					
Positive	476 (72)	240 (73)	75 (86)	97 (56)	28 (46)
Negative	147 (22)	78 (24)	12 (14)	74 (43)	20 (33)
Indeterminate	1 (0)	1 (0)	0	0 (0)	0
Unknown	36 (6)	10 (3)	0	2 (1)	13 (21)
**PR**					
Positive	411 (62)	206 (63)	69 (79)	81 (47)	22 (36)
Negative	210 (32)	111 (34)	18 (21)	90 (52)	26 (43)
Indeterminate	2 (0)	2 (0)	0	0 (0)	0
Unknown	37 (6)	10 (3)	0	2 (1)	13 (21)
**HER2**					
Positive	101 (15)	44 (13)	15 (17)	102 (59)	14 (23)
Negative	329 (50)	177 (54)	46 (53)	69 (40)	31 (51)
Equivocal	100 (15)	59 (18)	19 (22)	0 (0)	0
Unknown	130 (20)	49 (15)	7 (8)	2 (1)	16 (26)
**Histologic type**					
IDC	465 (70)	228 (69)	75 (86)	N/A	37 (60)
ILC	118 (18)	74 (23)	10 (12)	N/A	12 (20)
Other	77 (12)	26 (8)	2 (2)	N/A	12 (20)
Unknown	0 (0)	1 (0)	0	N/A	0
**Surgery type**					
Lumpectomy	147 (22)	76 (23)	22 (25)	N/A	27 (44)
Mastectomy	316 (48)	172 (53)	30 (35)	N/A	34 (56)
Other	157 (24)	67 (20)	35 (40)	N/A	0
Unknown	40 (6)	14 (4)	0	N/A	0
**Follow-up (y)**					
Median	2.20 (0.03-23.58)	2.22 (0.01-22.99)	3.93 (0.37-9.40)	3.91 (0.51-6.76)	5.39 (0.28-9.84)
Mean ± STD	3.40±3.34	3.39±2.25	3.99±2.22	3.85±1.46	4.77±2.749
**Recurrence**					
Event	N/A	N/A	N/A	49 (28)	23 (38)
No Event	N/A	N/A	N/A	124 (72)	38 (62)
Unknown	N/A	N/A	N/A	0 (0)	0
**Death**					
Event	101 (15)	50 (15)	1 (1)	32 (18)	N/A
No Event	559 (85)	279 (85)	86 (99)	138 (80)	N/A
Unknown	0	0	0	3 (2)	N/A

ER=Oestrogen receptor; PR=Progesterone receptor; HER2=Human epidermal growth factor receptor; IDC= Infiltrating ductal carcinoma; ILC= Infiltrating lobular carcinoma; N/A=Not applicable

Supplementary Table 4. Imaging feature description

Feature class	Feature description
Histogram distribution (n=10)	Mean, Standard Deviation, Kurtosis, Skewness, Maximum, Minimum, Median, Interquartile Range, Range, Entropy
Texture GLCM (n=19)	Energy, Contrast, Correlation, Sum of Squares Variance, Homogeneity, Sum Average, Sum Variance, Sum Entropy, Entropy, Difference Variance, Difference Entropy, Information Correlation 1, Information Correlation 2, Autocorrelation, Dissimilarity, Cluster Shade, Cluster Prominence, Maximum Probability, Inverse Difference
Morphologic (n=10)	Volume, Eccentricity, Surface Area, Compactness, Maximum Lesion Radius, Mean Radius of the Lesion, Radius of the Sphere, Compactness 1, Compactness 2, Surface Area/Volume

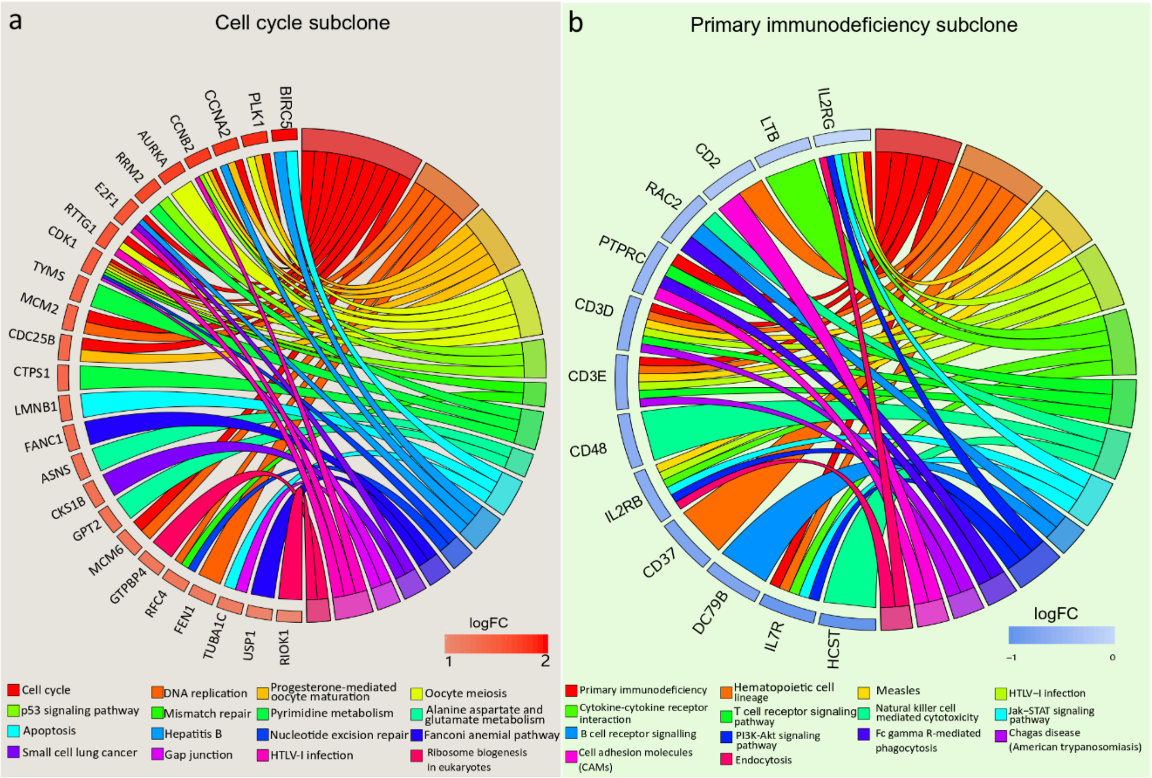

Supplementary Figure 1. Illustration of differentially expressed genes of subclones between the poor-survival and good-survival groups. Correlations among genes and corresponding biological functions for the **a)** ‘cell cycle’ and **b)** ‘primary immunodeficiency’ subclones are illustrated. Source data are provided as a Source data file.

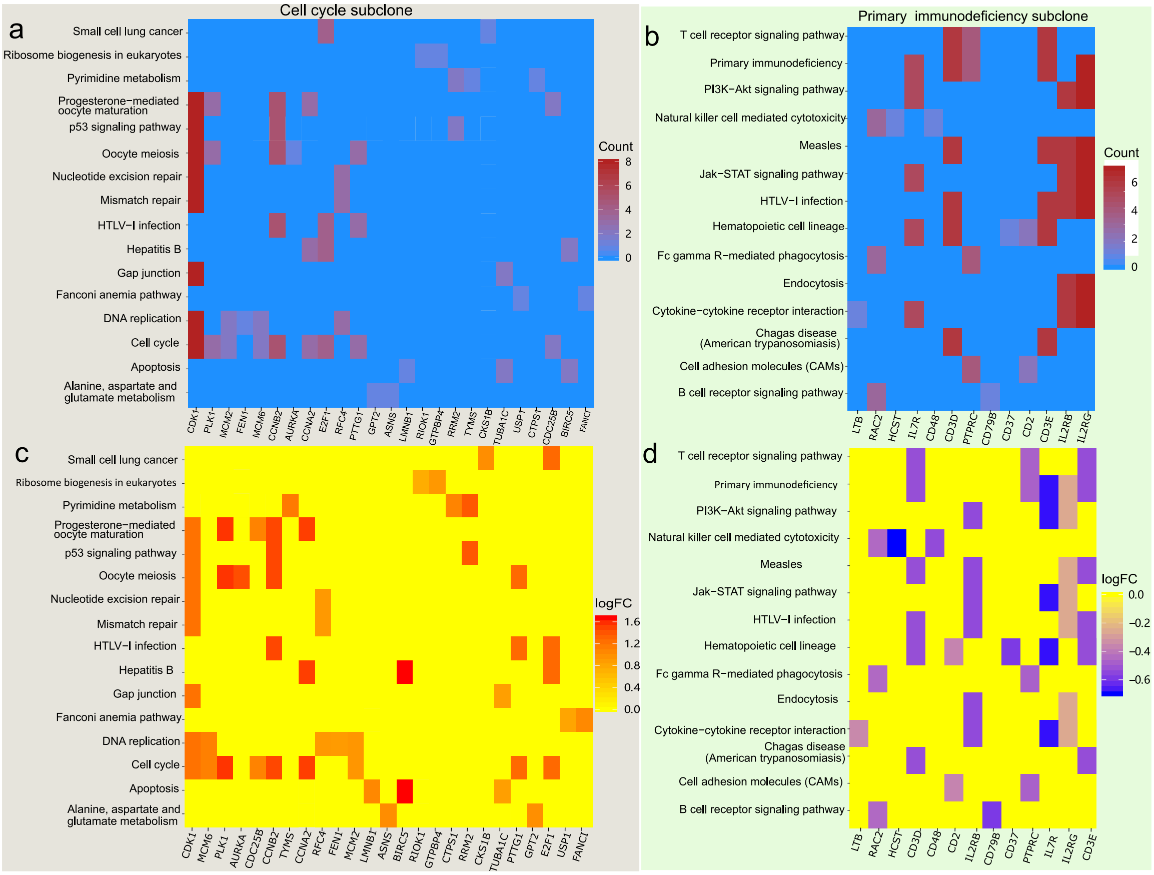

Supplementary Figure 2. Heatmap showing the relationship between genes (columns) and biological functions (rows). The colour of each cell represents the overall number of biological functions for the **a)** ‘cell cycle’ and **b)** ‘primary immunodeficiency’ subclones. The colour also reflects the logFC values for the **c)** ‘cell cycle’ and **d)** ‘primary immunodeficiency’ subclones. Source data are provided as a Source data file.
