# Supplementary material for: Radiogenomic signatures reveal multiscale intratumour heterogeneity associated with biological functions and survival in breast cancer
Source: Nat Commun. 2020 Sep 25;11:4861. doi: 10.1038/s41467-020-18703-2 (PMC7519071; doi:10.1038/s41467-020-18703-2)
Supplement: Supplementary file 3 — Reporting Summary [file 41467_2020_18703_MOESM3_ESM.pdf]

natureresearch

Corresponding author(s): Lihua Li and Yue Wang

Last updated by author(s): Sep 7, 2020

Reporting Summary

Nature Research wishes to improve the reproducibility of the work that we publish. This form provides structure for consistency and transparency in reporting. For further information on Nature Research policies, see Authors & Referees and the Editorial Policy Checklist .

Statistics

For all statistical analyses, confirm that the following items are present in the figure legend, table legend, main text, or Methods section.

n/a	Confirmed	
☐	☒	The exact sample size (*n*) for each experimental group/condition, given as a discrete number and unit of measurement
☐	☒	A statement on whether measurements were taken from distinct samples or whether the same sample was measured repeatedly
☐	☒	The statistical test(s) used AND whether they are one- or two-sided<br>*Only common tests should be described solely by name; describe more complex techniques in the Methods section.*
☐	☒	A description of all covariates tested
☐	☒	A description of any assumptions or corrections, such as tests of normality and adjustment for multiple comparisons
☐	☒	A full description of the statistical parameters including central tendency (e.g. means) or other basic estimates (e.g. regression coefficient) AND variation (e.g. standard deviation) or associated estimates of uncertainty (e.g. confidence intervals)
☐	☒	For null hypothesis testing, the test statistic (e.g. *F*, *t*, *r*) with confidence intervals, effect sizes, degrees of freedom and *P* value noted<br>*Give P values as exact values whenever suitable.*
☒	☐	For Bayesian analysis, information on the choice of priors and Markov chain Monte Carlo settings
☒	☐	For hierarchical and complex designs, identification of the appropriate level for tests and full reporting of outcomes
☒	☐	Estimates of effect sizes (e.g. Cohen's *d*, Pearson's *r*), indicating how they were calculated

Our web collection on statistics for biologists contains articles on many of the points above.

Software and code

Policy information about availability of computer code

Data collection	This is a retrospective study using pre-existing data, thus no software for data collection is involved.
Data analysis	Gene expression deconvolution software debCAM is publicly available for use [http://www.bioconductor.org/packages/release/bioc/html/debCAM.html].<br>Radiomic analysis was performed using the PET Oncology Radiomics Test Suite (PORTS) package available on website [https://www.mathworks.com/matlabcentral/fileexchange/55587-ports-3d-image-texture-metric-calculation-package].<br>The pyradiomics document and software are avaiable on website [https://pyradiomics.readthedocs.io/en/latest/].<br>Codes for genomic subclone identification and validation were available on Github [https://github.com/mfan0809/Gene-expression-deconvolution.git].<br>MATLAB (R2015, MathWorks, Natick, MA, USA) and R (version 4.0 R Foundation for Statistical Computing, Vienna, Austria) were used to analyze data in this study.

For manuscripts utilizing custom algorithms or software that are central to the research but not yet described in published literature, software must be made available to editors/reviewers. We strongly encourage code deposition in a community repository (e.g. GitHub). See the Nature Research guidelines for submitting code & software for further information.

Data

Policy information about availability of data

All manuscripts must include a data availability statement. This statement should provide the following information, where applicable:
- Accession codes, unique identifiers, or web links for publicly available datasets
- A list of figures that have associated raw data
- A description of any restrictions on data availability

The gene expression data of Cohort 1 (Genomic development and Genomic testing datasets) and Cohort 2 (Radiogenomic dataset) are available from the TCGA-BRCA project of Genomic Data Commons [https://portal.gdc.cancer.gov/projects/TCGA-BRCA].

The matched imaging data of Cohorts 1 and 2 are available from the TCGA-BRCA in TCIA: [https://wiki.cancerimagingarchive.net/display/Public/TCGA-BRCA].
The imaging, clinical and survival data of the Prognostic testing dataset are available from the Breast-MRI-NACT-Pilot in TCIA on website [https://wiki.cancerimagingarchive.net/display/Public/Breast-MRI-NACT-Pilot].
The imaging, clinical and survival data of the Prognostic assessment dataset are available from the ISPY-1 trail in TCIA on website [https://wiki.cancerimagingarchive.net/display/Public/ISPY1].
The source data used in Figs. 3a-b, 5a-b, 6a-c, and Supplementary Figs. 1a-b and 2a-d, and Table 1 are provided as a Source Data file. Source data are provided with this paper. The remaining data are available within the article, Supplementary Information or available from the author upon request.

Field-specific reporting

Please select the one below that is the best fit for your research. If you are not sure, read the appropriate sections before making your selection.

[x] Life sciences [ ] Behavioural & social sciences [ ] Ecological, evolutionary & environmental sciences

For a reference copy of the document with all sections, see nature.com/documents/nr-reporting-summary-flat.pdf

Life sciences study design

All studies must disclose on these points even when the disclosure is negative.

Sample size

All available datasets from TCGA and TCIA cohorts are outlined in the Methods, and the sample sizes are noted throughout the study.

Data exclusions

All available datasets were used where possible. Some data were excluded according to the pre-established exclusion criteria, as described in the Methods. For example, some samples in the genomic development and genomic validation datasets were excluded because there was no clinical report or insufficient survival data. For the radiogenomic dataset, samples were excluded due to 1) the variation in scan protocols; 2) absent gene expression data; 3) missing clinical information, and 4) incomplete DCE-MRI data.

Replication

The overall findings of this study were replicated in two datasets. The prognostic significance of genomic subclone was evaluated and validated in two datasets from TCGA cohort that contained both gene expression and survival data. The prognostic value of the identified radiogenomic signatures was assessed and validated in two additional independent datasets that contained dynamic contrast-enhanced MR imaging (DCE-MRI) and survival outcome data.

Randomization

Our work is a retrospective study (data analyses) using pre-existing data from TCGA and TCIA, so we could not control how samples were allocated into experimental groups in TCIA/TCGA. Description on how samples were allocated into experimental groups from TCIA/TCGA is presented at URLs provided in the Data availability. In our study, we randomly separated the dataset (Cohort 1) from the TCGA into a genomic development dataset (n=660, 66.7%) and a genomic validation dataset (n=329, 33.3%). Cohort 2 (radiogenomic dataset, n=87) contains matched imaging and genomic data, where the genomic data were obtained from TCGA-BRCA and the matched imaging data were obtained from TCIA. Because this cohort is used only for an association analysis, no randomization is needed. Cohort 3 contains matched DCE-MRI and follow-up data and includes two independent datasets, the prognostic assessment dataset and the prognostic testing dataset, which were collected from the ISPY-1 trials and the Breast-NACT-pilot respectively in TCIA. Again, as a retrospective study, we could not control how samples were allocated into these cohorts or sub-cohorts.

Blinding

Blinding is not relevant to this study for the following reasons. Our study does not involve manipulating samples for different groups. No reference information of samples is needed. All samples were treated in the same way, and the experiments do not rely on human interpretation. The carefully performed image processing, bioinformatics, and statistical analyses are considered unbiased when applied to the datasets used in this study.

Reporting for specific materials, systems and methods

We require information from authors about some types of materials, experimental systems and methods used in many studies. Here, indicate whether each material, system or method listed is relevant to your study. If you are not sure if a list item applies to your research, read the appropriate section before selecting a response.

Materials & experimental systems

n/a	Involved in the study	
[x]	[ ]	Antibodies
[x]	[ ]	Eukaryotic cell lines
[x]	[ ]	Palaeontology
[x]	[ ]	Animals and other organisms
[x]	[ ]	Human research participants
[x]	[ ]	Clinical data

Methods

n/a	Involved in the study	
[x]	[ ]	ChIP-seq
[x]	[ ]	Flow cytometry
[x]	[ ]	MRI-based neuroimaging
